# Efficacy and safety of immune checkpoint inhibitors in colorectal cancer: a systematic review and meta-analysis

**DOI:** 10.1007/s00384-021-04028-z

**Published:** 2021-10-29

**Authors:** Tianni Zeng, Xiaojie Fang, Jinhua Lu, Yazhen Zhong, Xianlei Lin, Zechen Lin, Nan Wang, Jing Jiang, Shengyou Lin

**Affiliations:** 1 Department of Oncology, Hangzhou TCM hospital Affiliated to Zhejiang Chinese Medical University, Hangzhou, China; 2 Department of Anorectal Surgery, Hangzhou TCM hospital Affiliated to Zhejiang Chinese Medical University, Hangzhou, China; 3 The Third Clinical Medical College, Zhejiang Chinese Medical University, Hangzhou, China; 4 The First People’s Hospital of Xiaoshan District, Hangzhou, China

**Keywords:** Programmed death ligand 1 inhibitor, Programmed cell death protein 1 inhibitor, Immune checkpoint inhibitors, Colorectal cancer, Efficacy, Safety

## Abstract

**Background and objective:**

Immune checkpoint inhibitor (ICI) therapies have shown promising prospects in colorectal cancer (CRC) immunotherapy; many clinical trials have been carried out. In this study, we sought to evaluate the efficacy and safety of ICI therapies in CRC by presenting a meta-analysis of relevant studies.

**Methods:**

Databases including PubMed, Embase, Cochrane Library, and Web of Science were systematically searched for studies concerning the efficacy and safety of ICI in colorectal cancer. The reported odds ratio (*OR*) or weighted mean difference (*WMD*) with 95% confidence intervals (*CI*s) of overall survival (*OS*), progression-free survival (*PFS*), objective response rate (*ORR*), disease control rate (*DCR*), treatment-related adverse events (*TRAE*s), and *TRAE*s ≥ 3 in the included studies were analyzed by fixed effects/random effects models.

**Results:**

Three studies involving 667 patients with colorectal cancer were included in our meta-analysis. No significant difference between the immune checkpoint inhibitor therapies and conventional therapies in *OS* (*WMD* = 0.73, 95% *CI* − 3.09, 4.54; *p* = 0.71), in *ORR* (*OR* = 1.54, 95% *CI* 0.98, 2.40; *p* = 0.06), and in *DCR* (*OR* = 0.97, 95% *CI* 0.36, 2.61; *p* = 0.95). The median *PFS* of the ICI therapy group was shorter than that of the conventional therapy group (*WMD* =  − 0.10, 95% *CI* − 0.18, − 0.02; *p* = 0.02). At the same time, we also could not find a significant difference between the immune checkpoint inhibitor therapies and conventional therapies in *TRAE*s (*OR* = 1.56, 95% *CI* 0.11, 22.09; *p* = 0.74) and in *TRAE*s ≥ 3 (*OR* = 0.94, 95% *CI* 0.16, 5.65; *p* = 0.95).

**Conclusion:**

Immune checkpoint inhibitor therapies could not improve all survival endpoints to advanced or metastatic colorectal cancer patients. Whether immune checkpoint inhibitors should be the first choice of therapies for colorectal cancer patients with undetermined microsatellite status or not able to determine microsatellite status needs more related studies to prove.

## Introduction

Globally, colorectal cancer is the fourth most fatal cancer, with approximately 0.9 million annual mortalities. It is the second most common tumor among women and the third most common tumor among men [1]. Colorectal cancer accounts for about 10% of all annually diagnosed cancers and cancer-related mortalities [2]. Morbidity and mortality rates are highest in the developed countries. By the year 2035, annual global incidences of colorectal cancer are predicted to increase to 2,500,000 [3].

Since clinical symptoms of colorectal cancer occur late, most patients are diagnosed when the disease is in the advanced stages [1]. Depending on cancer characteristics, first-line therapy for advanced CRC is combination chemotherapy plus an anti-epidermal growth factor receptor (EGFR) antibody or anti-vascular endothelial growth factor (VEGF) [4, 5]; however, progression-free survival time for most patients is within 9–12 months [6–13]. Therefore, there is a need to develop effective therapeutic options with fewer side effects.

Immunotherapy has improved tumor treatment [14]. In this context, metastatic colorectal cancer represents an intriguing entity, with a minority (4–5%) of tumors which harbor microsatellite instability (MSI) and/or mismatch repair deficiency (dMMR) being highly sensitive to immune checkpoint inhibitors, while a vast majority of immunologically “cold” tumors are refractory to immunotherapeutic strategies [15]. It has been confirmed that immune checkpoint inhibitors play an important role in colorectal cancer patients that are mismatch repair deficient (dMMR) [16, 17]. Some humanized monoclonal antibodies, including ipilimumab, nivolumab, pembrolizumab, avelumab, atezolizumab, and durvalumab among others, have been developed. These drugs have been approved for use in malignant cancers such as melanoma, non–small cell lung cancer, urothelial carcinoma, Hodgkin’s lymphoma, and head and neck squamous carcinoma among others [18]. The National Comprehensive Cancer Network guidelines recommend pembrolizumab or nivolumab as second-line therapeutic options especially for patients with dMMR/MSI-high mCRC [19]. However, questions remain concerning the role of immune checkpoint inhibitors (ICIs) for the treatment of microsatellite-stable (MSS) and mismatch repair–proficient (pMMR) CRC. It has not been established if the efficacy of immune checkpoint inhibitors is superior to that of other therapies for colorectal cancer, whether with pMMR or dMMR. Therefore, we performed a meta-analysis of various clinical trials involving colorectal cancer to evaluate the safety and efficacy of immune checkpoint inhibitors.

## Methods

The systematic review protocol for this study was registered in PROSPERO (registration number CRD42021238819).

### Literature search strategies

Electronic databases, including PubMed, Embase, Cochrane Library, and Web of Science, were searched. We searched the literature from inception to March 2021 without restriction of language. In our search strategy, the MeSH terms combined with related words and keywords were adjusted to comply with the relevant rules in each database. Search terms included “colorectal neoplasm,” “colorectal tumor,” “b7 h1 antigen,” “cd274 antigen,” “programmed cell death protein 1 inhibitor,” “programmed death ligand 1 inhibitor,” “immune checkpoint inhibitors,” “nivolumab,” “pembrolizumab,” “atezolizumab,” “durvalumab,” “avelumab,” and “ipilimumab.” All entries that satisfied these criteria were manually retrieved.

### Inclusion and exclusion criteria

The inclusion criteria for eligible studies were (i) confirmed diagnosis of colorectal cancer; (ii) data on overall survival (*OS*) or progression-free survival (*PFS*) were available for evaluating the efficacy of immune checkpoint inhibitors; (iii) security indicators, including treatment-related adverse events (*TRAE*s), grade ≥ 3 *TRAE*s were directly provided or could be calculated; and (iv) RCTs, irrespective of the blinding method or lack of, were also included. Animal studies, reviews, editorials, comments, meetings, or case reports were excluded. Studies with duplicate publications, unbalanced matching procedures, or incomplete data were also excluded.

### Data extraction

Two reviewers (Fang xj and Lin xl) independently extracted the data according to the prescribed selection criteria. Differences in opinion were resolved by discussion between the authors or by obtaining an opinion from a third evaluator. The following data were extracted: the name of the first author, year of publication, number of patients, study design, age, gender, tumor types, previous treatment, organ status, Eastern Cooperative Oncology Group (ECOG) performance status, intervention methods, and statistical data including *OS*, *PFS*, *ORR*, *DCR*, *TRAE*s, and grade ≥ 3 *TRAE*s. Where necessary, corresponding authors were contacted to obtain supplementary information.

### Quality assessment

**Fig.1:**
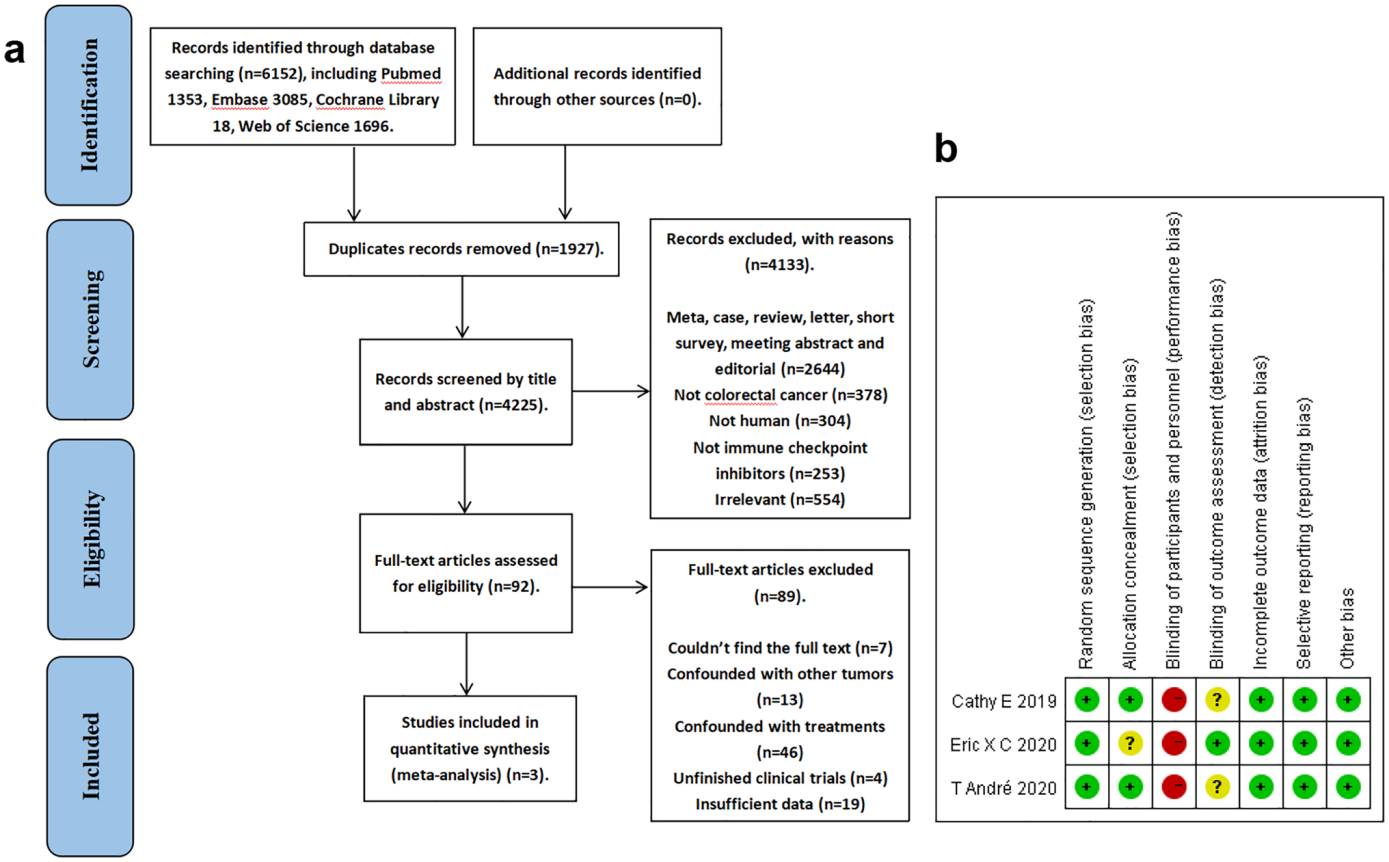
**a** Flowchart of the study selection process. **b** Risk of bias summary of randomized controlled trials. + low risk, ? unclear risk, − high risk

Cochrane Collaboration’s tool for assessing risk of bias was used for assessing the quality of each included study (Fig. 1b). For evaluating selection bias, performance bias, detection bias, attrition bias, reporting bias, and other bias, there were seven items provided by the tool. They contained random sequence generation, allocation concealment, blinding of participants and personnel, blinding of outcome assessment, incomplete outcome data, selective reporting, and other bias. Each item was answered with one of the three replies: low risk, unclear risk, and high risk to assess the bias.

### Statistical analysis

Data were extracted from the primary studies and analyzed using Review Manager (version 5.3, Cochrane Collaboration, Oxford, UK). We expressed results for dichotomous outcomes as odd risk with 95% confidence intervals (*CI*s) and continuous outcomes as weighted mean difference. In the absence of statistical heterogeneity, a fixed effects model was used to pool data. In cases of statistical heterogeneity (*p* < 0.05, *I*^2^ ≥ 50%), a random effects model was used.

## Results

### Search results

From the systematic database search, we retrieved 6152 articles. Based on the inclusion criteria, at total of 6149 articles were excluded, with 3 [20–22] articles being eligible for the meta-analysis (Fig. 1a). From the included studies, a total of 667 patients were enrolled. Characteristics of all included studies are shown in Table 1.

**Table 1:** Main characteristics of included studies

Study author (year)	Study design	Gender (M/F)	Case Experimental vs control	Patients’ characteristics	Intervention methods
Eng et al. [20]	RCT phase 3	70/110	180 90 vs 90	Advanced or metastatic colorectal cancer; disease progression on or intolerance to at least two previous systemic chemotherapy regimens (containing fluorouracil, oxaliplatin, and irinotecan) was enrolled; received previous anti-angiogenic or anti-epidermal growth factor receptor (EGFR) therapy were eligible; adequate hematological and end organ function; ECOG 0–1; ≥ 18 years old	Atezolizumab (PD-L1) 1200 mg/3 weeks ivgtt vs regorafenib 160 mg po d1–21/4 weeks
Chen et al. [21]	RCT phase 2	121/59	180 119 vs 61	Histologically confirmed adenocarcinoma of the colon or rectum; received all available standard systemic therapies fluoropyrimidines, oxaliplatin, irinotecan, and bevacizumab if appropriate; cetuximab or panitumumab if RAS wild-type tumors; regorafenib if available; measurable disease; adequate organ function; ECOG 0–1; ≥ 18 years old	Durvalumab (PD-L1) 1500 mg/4 weeks ivgtt plus tremelimumab (CTLA-4) 75 mg/4 weeks for the initial 4 cycles only ivgtt plus best supportive care vs best supportive care
André et al. [22]	RCT phase 3	153/154	307 153 vs 154	MSI-H–dMMR stage IV colorectal cancer; received previous adjuvant chemotherapy for colorectal cancer if the earlier treatment had been completed at least 6 months before randomization; measurable disease according to Response Evaluation Criteria in Solid Tumor (RECIST), version 1.1; adequate organ function; ECOG 0–1; ≥ 18 years old	Pembrolizumab (PD-1) 200 mg/3 weeks ivgtt vs mFOLFOX6 (oxaliplatin 85 mg/m^2^ ivgtt d1/2 weeks plus leucovorin 400 mg/m^2^ ivgtt d1/2 weeks plus 5-fluoropyrimidine 400 mg/m^2^ ivgtt d1/2 weeks and 1200 mg/m^2^ ivgtt d2,3/2 weeks) or FOLFOX6 plus bevacizumab 5 mg/kg ivgtt d1/2 weeks or mFOLFOX6 plus cetuximab 400 mg/m^2^ ivgtt over 2 h (first infusion) followed by 250 mg/m^2^/week ivgtt or FOLFIRI (irinotecan 180 mg/m^2^ ivgtt d1/2 weeks plus leucovorin 400 mg/m^2^ ivgtt d1/2 weeks plus 5-fluoropyrimidine 400 mg/m^2^ ivgtt d1/2 weeks and 1200 mg/m^2^ ivgtt d2,3/2 weeks) or FOLFIRI plus bevacizumab or FOLFIRI plus cetuximab (with bevacizumab and cetuximab administered at the same doses as those listed above with mFOLFOX6)

### Meta-analysis

#### Overall survival (OS)

**Fig. 2:**
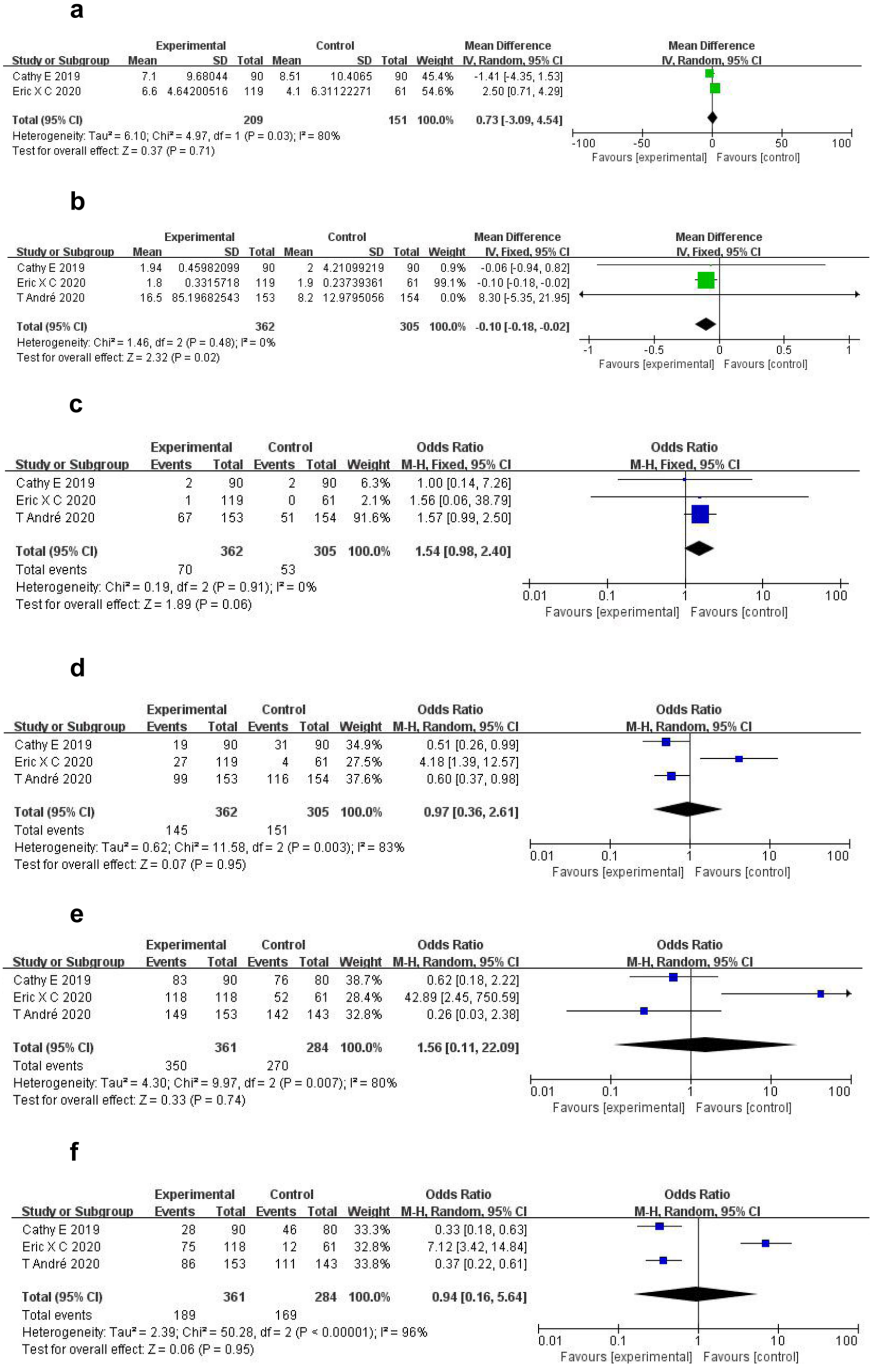
Forest plots of different subgroups. **a** *OS* time. **b** Median *PFS* time. **c** *ORR*. **d** *DCR*. **e** *TRAE*s. **f** *TRAE*s ≥ 3. *CI* confidence interval, *OS* overall survival, *PFS* progression-free survival, *ORR* objective response rate, *DCR* disease control rate, *TRAE*s treatment-related adverse events, *OR* odd risk, *WMD* weighted mean difference

Data on median overall survival outcomes for the 360 CRC patients were obtained. There was no significant difference between the outcomes of immune checkpoint inhibitor therapy and conventional therapy (*WMD* = 0.73, 95% *CI* − 3.09, 4.54; *p* = 0.71). Significant heterogeneity was observed in these studies (*p* = 0.03; *I*^2^ = 80%; Fig. 2a).

#### Progression-free survival (PFS)

For the 667 CRC patients, the median progression-free survival time of the immune checkpoint inhibitor therapy group was significantly shorter than that of the conventional therapy group (*WMD* =  − 0.10, 95% *CI* − 0.18, − 0.02; *p* = 0.02). Significant heterogeneity was not observed in these studies (*p* = 0.48; *I*^2^ = 0%; Fig. 2b).

#### Objective response rate (ORR)

There was no significant difference in *ORR* between the two groups (*OR* = 1.54, 95% *CI* 0.98, 2.40; *p* = 0.06). Significant heterogeneity was not observed in these studies (*p* = 0.91; *I*^2^ = 0%; Fig. 2c).

#### Disease control rate (DCR)

There was no significant difference in *DCR* between the immune checkpoint inhibitor therapy group and the conventional therapy group (*OR* = 0.97, 95% *CI* 0.36, 2.61; *p* = 0.95). Significant heterogeneity was found in these studies (*p* = 0.003; *I*^2^ = 83%; Fig. 2d).

#### Treatment-related adverse events (TRAEs)

Incidences of *TRAE*s in the immune checkpoint inhibitor therapy group and conventional therapy group were 97.0% and 95.1%, respectively, a difference that was not significant (*OR* = 1.56, 95% *CI* 0.11, 22.09; *p* = 0.74). Significant heterogeneity was observed in these studies (*p* = 0.007; *I*^2^ = 80%; Fig. 2e). Incidences of *TRAE*s ≥ 3 in the immune checkpoint inhibitor therapy and conventional therapy groups were 52.4% and 59.5%, respectively, a difference that was not significant (*OR* = 0.94, 95% *CI* 0.16, 5.65; *p* = 0.95). Significant heterogeneity was observed in these studies (*p* < 0.00001; *I*^2^ = 96%; Fig. 2f).

## Discussion

This is the first meta-analysis to evaluate the efficacy and safety of immune checkpoint inhibitors, including PD-1, PD-L1, and CTLA-4 antibodies, as therapeutic options for colorectal cancer. We found that differences in overall survival outcomes were not significant between the immune checkpoint inhibitor group and the group with other therapeutic options (including chemotherapy and best supportive care). Treatment with immune checkpoint inhibitors was associated with poor progression-free survival. In objective response and disease control rates, there was no significant difference between the two groups. More than half of patients in the immune checkpoint inhibitor therapy group exhibited ≥ 3*TRAE*s, with the incidence in the immune checkpoint inhibitor therapy group being lower than that of the conventional therapy group. However, *OR*s for *TRAE*s and ≥ 3*TRAE*s were not significantly different between the immune checkpoint inhibitor therapy and the conventional therapy groups. ICI therapy did not exhibit any particular advantage over conventional therapy in advanced CRC patients. Moreover, incidences of adverse events in the ICI therapy group were not significantly lower than those of the conventional therapy group in advanced CRC patients.

It has been shown that compared to chemotherapy, immune checkpoint inhibitors exhibit unique response and survival outcomes for patients with advanced mismatch repair–deficient/microsatellite-unstable (dMMR/MSI) colorectal cancer, but have shown disappointing results in mismatch repair–proficient/microsatellite-stable (pMMR/MSS) colorectal cancer patients [15, 19, 23]. Negative results could be attributed to the lack of comparisons of the efficacy and safety of immune checkpoint inhibitors in dMMR/MSI colorectal cancer and pMMR/MSS colorectal cancer. Currently, the number of RCTs evaluating the efficacy and safety of immune checkpoint inhibitors in dMMR/MSI colorectal cancer and pMMR/MSS colorectal cancer is very small. More RCTs are needed to confirm our results.

This study is associated with some limitations. First, we only used three RCTs. The sample size was relatively small. Second, we generally analyzed dMMR/MSI and pMMR/MSS colorectal cancer patients, and we did not perform subgroup analysis based on colorectal cancer microsatellite status. Studies should aim at evaluating whether the efficacy and safety of immune checkpoint inhibitors change according to the microsatellite status of CRC. Finally, despite applications of the random effects model, there was substantive heterogeneity in some of the results. We could not use subgroup and sensitivity analyses because the number of the included studies was relatively small.

## Conclusion

Immune checkpoint inhibitor therapies have no particular advantage over non-immune checkpoint inhibitor therapies. Moreover, incidences of adverse events due to immune checkpoint inhibitor therapy are not significantly lower than those of non-immune checkpoint inhibitor therapy. Therefore, under the existing evidence, immune checkpoint inhibitors should not be first choice therapies for colorectal cancer patients with undetermined microsatellite status.
